# Bacteria in cancer therapy: a novel experimental strategy

**DOI:** 10.1186/1423-0127-17-21

**Published:** 2010-03-23

**Authors:** S Patyar, R Joshi, DS Prasad Byrav, A Prakash, B Medhi, BK Das

**Affiliations:** 1 Department of Pharmacology, Post Graduate Institute of Medical Education and Research, Chandigarh 160012, India; 2 Department of Microbiology, All India Institute of Medical Sciences, New Delhi 110029, India

## Abstract

Resistance to conventional anticancer therapies in patients with advanced solid tumors has prompted the need of alternative cancer therapies. Moreover, the success of novel cancer therapies depends on their selectivity for cancer cells with limited toxicity to normal tissues. Several decades after Coley's work a variety of natural and genetically modified non-pathogenic bacterial species are being explored as potential antitumor agents, either to provide direct tumoricidal effects or to deliver tumoricidal molecules. Live, attenuated or genetically modified non-pathogenic bacterial species are capable of multiplying selectively in tumors and inhibiting their growth. Due to their selectivity for tumor tissues, these bacteria and their spores also serve as ideal vectors for delivering therapeutic proteins to tumors. Bacterial toxins too have emerged as promising cancer treatment strategy. The most potential and promising strategy is bacteria based gene-directed enzyme prodrug therapy. Although it has shown successful results *in vivo *yet further investigation about the targeting mechanisms of the bacteria are required to make it a complete therapeutic approach in cancer treatment.

## Review

Cancer is characterized by uncontrolled and invasive growth of cells. These cells may spread to other parts of the body, and this is called metastasis. Although conventional anticancer therapies, consisting of surgical resection, radiotherapy and chemotherapy, are effective in the management of many patients but for about half of cancer sufferers these are ineffective, so alternative techniques are being developed to target their tumours. Experimental cancer treatments are medical therapies intended or claimed to treat cancer by improving, supplementing or replacing conventional methods. These include photodynamic therapy, HAMLET (human alpha-lactalbumin made lethal to tumor cells), gene therapy, telomerase therapy, hyperthermia therapy, dichloroacetate (DCA), non-invasive RF cancer treatment, complementary and alternative therapy, diet therapy, insulin potentiating therapy and bacterial treatment [1]. But many of these therapies are controversial due to lack of evidence, efficacy, feasibility, availability, specificity and selectivity. It has been reported that some microorganisms display selective replication in tumor cells or preferential accumulation in the tumor micro-environment thus offering a great potential for cancer therapy. Many viruses, like vaccinia virus, Newcastle disease virus, reovirus and adenovirus with an E1a deletion, which are intended to achieve selective replication and killing of tumor cells have been investigated. Viruses have shown the most potential to carry altered genes to cancer cells, to find target cells in body and ability to latch onto these cells. Oncolytic viruses cause lysis (rupture) of cancer cells, which can then be processed by the adaptive immune system, which can then target similar cells in other parts of the body. But the effective use of such viruses is sometimes hindered by the production of potentially neutralizing antibodies generated against them [2]. It has been reported that some bacterial species also preferentially replicate and accumulate within tumors. Moreover, they possess certain advantageous features such as motility, capacity to simultaneously carry and express multiple therapeutic proteins, and elimination by antibiotics, thus making bacterial treatment a promising new strategy in cancer treatment [3]. This review highlights the use of bacteria in cancer therapy as a novel experimental strategy.

## Background

The role of bacteria as anticancer agent was recognized almost hundred years back. The German physicians W. Busch and F. Fehleisen separately observed that certain types of cancers regressed following accidental erysipelas (*Streptococcus pyogenes*) infections that occurred whilst patients were hospitalized [4]. Independently, the American physician William Coley noticed that one of his patients suffering from neck cancer began to recover following an infection with erysipelas. He began the first well-documented use of bacteria and their toxins to treat end stage cancers. He developed a safer vaccine in the late 1800's composed of two killed bacterial species, *S. pyogenes *and *Serratia marcescens *to simulate an infection with the accompanying fever without the risk of an actual infection [5,6]. And the vaccine was widely used to successfully treat sarcomas, carcinomas, lymphomas, melanomas and myelomas. Complete, prolonged regression of advanced malignancy was documented in many cases [7]. Toxic bacterial derivatives 'Coley's toxins' were also studied for potential anticancer activity [8]. The early success of Coley's toxins provided the grounds for current advances in this field.

## Bacterial therapy

**Figure 1:**
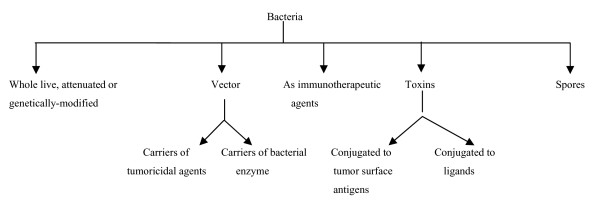
**Schematic overview of role of bacteria in cancer therapy**.

After Coley's initial observations, scientists discovered that certain species of anaerobic bacteria, such as those belonging to the genus *Clostridium*, thrive and consume oxygen-poor cancerous tissue whereas die when they come in contact with the tumor's oxygenated sides, meaning they would be harmless to the rest of the body [9]. These findings provided the rationale for using the bacteria as oncolytic agents. However, bacteria don't consume all parts of the malignant tissue thus underlying the need of combining the therapy with chemotherapeutic treatments. Thus bacteria can be implied as sensitising agents for chemotherapy. Bacterial products like endotoxins (Lipopolysaccharides) have to some extent already been tested for cancer treatment. Bacterial toxins can be used for tumor destruction and cancer vaccines can be based on immunotoxins of bacterial origin [10]. Bacteria can be exploited as delivery agents for anticancer drugs, and as vectors for gene therapy. Spores of anaerobic bacteria can be used for the aforementioned strategies because only spores that reach an oxygen starved area of a tumour will germinate, multiply and become active. The use of genetically modified bacteria for selective destruction of tumors, and bacterial gene-directed enzyme prodrug therapy have shown promising potential. The detailed overview of these bacteria based approaches is given below (Fig. 1).

## Bacteria as tumoricidal agents

The use of live, attenuated or genetically-modified, non-pathogenic bacteria has begun to emerge as potential antitumor agents, either to provide direct tumoricidal effects or to deliver tumoricidal molecules. Experimental studies have shown that pathogenic species of the anaerobic clostridia were able to proliferate preferentially within the necrotic (anaerobic) regions of tumors in animals as compared to normal tissues thus resulting in tumour regression but was accompanied by acute toxicity and most animals became ill or died [9,11]. This shifted the focus to a non-pathogenic strain of *Clostridium *such as 'M55', showing that it was able to colonize anaerobic parts of the tumour following intravenous administration but did not produce significant tumour regression [12]. Recently, a number of anaerobic bacterial species (bifidobacteria, lactobacilli and pathogenic clostridia) have been screened for their ability to accumulate in experimental tumors in animals. *Clostridium novyi *demonstrated significant anti-tumor effects, but these experiments too culminated in death. An attenuated strain known as *C. novyi-NT *was obtained after deleting a gene coding for a lethal toxin exhibited good results but produced toxicity also.

Thus, *C. novyi-NT *spores were administered in combination with conventional chemotherapeutic agents like dolastatin-10, mitomycin C, vinorelbine and docetaxel. This strategy known as combination bacteriolytic therapy (COBALT) resulted in significant anti-tumour properties but still was not devoid of animal deaths [13]. *C. novyi *has also been investigated in conjunction with radiotherapy, radioimmunotherapy, and further chemotherapy in experimental tumor models [14,15]. The results have demonstrated the potential of combined multi-modality approaches as developmental future cancer therapies. *C. novyi-NT *has been exploited to enhance the release of liposome-encapsulated drugs within tumors because of its evident membrane-disrupting potential. The bacterial factor responsible for the enhanced drug release has been identified as liposomase. Remarkable eradication of the tumors in mice bearing large, established tumors by employing *C. novyi-NT *plus a single dose of liposomal doxorubicin has led to further studies in the field [16]. To make the poorly vascularized regions of tumors accessible to drugs, *C. novyi-NT *was used in combination with anti-microtubule agents. Results demonstrated that the microtubule destabilizers such as HTI-286 and vinorelbine, but not the microtubule stabilizers such as the taxanes, docetaxel and MAC-321, radically reduced blood flow to tumors thereby enlarging the hypoxic region favourable for spores' germination [17]. Bacillus Calmette-Guerin (BCG), the most successful bacterial agent so far is used specifically for the treatment of superficial bladder cancer. VNP20009, a derivative strain of *Salmonella typhimurium *has now been developed for use in cancer treatment. Deletion of two of its genes - *msbB *and *purI *-resulted in its complete attenuation (by preventing toxic shock in animal hosts) and dependence on external sources of purine for survival. This dependence renders the organism incapable of replicating in normal tissue such as the liver or spleen, but still capable of growing in tumours where purine is available. This vector showed long-lasting efficacy against a broad range of experimental tumors and was even able to target metastatic lesions [18,19]. One advantage of using Salmonella instead of Clostridium or Bifidobacterium is its ability to grow in both aerobic and anaerobic conditions, indicating its usefulness against small tumors. VNP20009 has been investigated successfully in Phase 1 clinical trials in cancer patients. It is also likely that other live, attenuated bacteria, such as Clostridia and Bifidobacterium, will be evaluated in human clinical trials in the future. New strains of bacteria being investigated as anticancer agents are: *Salmonella choleraesuis*, *Vibrio cholerae*, *Listeria monocytogenes *and even *Escherichia coli *[20].

## Bacteria as vector for gene therapy

The major problem with using bacteria as anti-cancer agents is their toxicity at the dose required for therapeutic efficacy and reducing the dose results in diminished efficacy. And the basic obstacle in cancer gene therapy is the specific targeting of therapy directly to a solid tumor. One approach to overcome these limitations has been the use of bacteria, genetically engineered to express a specific therapeutic gene. By producing the protein of interest specifically in the tumor micro-environment, these bacterial vectors can provide a powerful adjuvant therapy to various cancer treatments. Thus bacteria serve as vectors or vehicles for preferentially delivering anticancer agents, cytotoxic peptides, therapeutic proteins or pro-drug converting enzymes to solid tumours.

### Bacteria as carriers of tumoricidal agents

A cya/crp (genes encoding proteins involved in the regulation of cyclic AMP levels) mutant of *S*. *typhimurium*, ×4550, has been engineered to express interleukin-2 for the treatment of liver cancer in preclinical models [21,22]. Since *S*. *typhimurium*, naturally colonizes in liver, it is hypothesized that its attenuated form could be used to deliver cytokines locally to liver, with an effect on hepatic metastases. Various therapeutic proteins, including TNF-α and platelet factor 4 fragment, have been cloned and expressed in VNP20009 [23,24]. hIL-12, hGM-CSF, mIL-12 and mGM-CSF, have been cloned under the control of a cytomegalovirus (CMV) promoter, into SL3261, an auxotrophic *S. typhimurium*. It was found that oral administration of Salmonella expressing either mGM-CSF or mGM-CSF plus mIL12 caused tumor regression in mice bearing Lewis lung carcinomas [25]. Functional TNF-α has been cloned and expressed in *C. acetobutylicum*. *Bifidobacterium adolescentis *has recently been used as a delivery system for the antiangiogenic protein endostatin. Systemic administration of its spores via tail vein of tumor-bearing mice resulted in a strong inhibition of angiogenesis and reduced tumor growth [26].

### Bacterially directed enzyme prodrug therapy

This strategy overcomes the unacceptable side effects of bacterial therapy and uses anaerobic bacteria that have been transformed with an enzyme that can convert a non-toxic prodrug into a toxic drug. With the proliferation of the bacteria in the necrotic and hypoxic areas of the tumor, the enzyme is expressed solely in the tumor. Thus a systemically applied prodrug is metabolized to the toxic drug only in the tumor [27]. Several enzyme/prodrug systems are available. Cytosine deaminase (CD), which converts 5-fluorocytosine (5FC) to 5-fluorouracil (5FU), and nitroreductase (NR), which converts the prodrug CB1954 to a DNA cross-linking agent, have been tested with *Clostridium sporogenes*. Although these combinations can kill tumor cells *in vitro *and deliver high concentrations of enzymes to model tumours, to date, the results *in vivo *have been disappointing. Similarly, CD expressed in *Clostridium acetobutylicum *has demonstrated a selective delivery of the active exogenous enzyme into tumors [28,29]. Recently it was demonstrated that CD can be successfully cloned and expressed in the same strain of Clostridium, and CD expression was enhanced significantly by the vascular targeting agent combretastatin A-4 phosphate. The enhancement may be due to the enlargement of the necrotic area in tumors [28].

The *Salmonella *vector has also been combined with NR and CD, and success has been observed *in vivo*. And both are currently undergoing phase I clinical trials in cancer patients. TAPET (Tumour Amplified Protein Expression Therapy) uses VNP20009, an attenuated strain of *S. typhimurium *as a bacterial vector and expresses an *E. coli *CD for preferentially delivering anticancer drugs to solid tumours [30]. The expression of the prodrug-converting enzyme HSV-thymidine kinase (TK) in a purine auxotrophe has demonstrated enhanced antitumor activity upon the addition of ganciclovir, the corresponding prodrug [31]. Expression of HSV-TK in VNP20009 has demonstrated its selective accumulation in subcutaneously implanted murine colon 38 tumors [32]. *Salmonella *has been combined with carboxypeptidase G2 (CPG2), an enzyme that converts a range of mustard prodrugs to DNA cross-linking agents. High levels of activity have been detected in tumours following *in vivo *administration prompting further research. For significant efficacy, both the prodrug and the activated drug must be able to cross biological membranes, because the prodrug will be activated within bacterial cells and the active drug will then need to enter the tumor cells. Transfected *B. longum *by pBLES100-S-eCD produces cytosine deaminase in the hypoxic tumor, and studies have confirmed this as an effective prodrug-enzyme therapy [33].

## Bacterial toxins for cancer treatment

Bacterial toxins have to some extent already been tested for cancer treatment. Bacterial toxins can kill cells or at reduced levels alter cellular processes that control proliferation, apoptosis and differentiation. These alterations are associated with carcinogenesis and may either stimulate cellular aberrations or inhibit normal cell controls. Cell-cycle inhibitors, such as cytolethal distending toxins (CDTs) and the cycle inhibiting factor (Cif), block mitosis and are thought to compromise the immune system by inhibiting clonal expansion of lymphocytes. In contrast, cell-cycle stimulators such as the cytotoxic necrotizing factor (CNF) promote cellular proliferation and interfere with cell differentiation [34]. Bacterial toxins that subvert the host eukaryotic cell cycle have been classified as cyclomodulins. For example, CNF is a cell-cycle stimulator released by certain bacteria, such as *E. coli*. CNF triggers G_1_- S transition and induces DNA replication. The number of cells does not increase, however. The cells become multinucleated instead, perhaps by the toxin's ability to inhibit cell differentiation and apoptosis [35,36]. CDTs are found in several species of Gram-negative bacteria, including *Campylobacter jejuni *and *S. typhi *while Cif is found in enteropathogenic (EPEC) and enterohaemorrhagic (EHEC) *E. coli*. The anti-tumor effect of toxins is probably with reduced side-effects compared to traditional tumor treatment. Bacterial toxins *per se *or when combined with anti-cancer drugs or irradiation could therefore possibly increase the efficacy of cancer treatment [10].

### Bacterial toxins binding to tumor surface antigens

Diphtheria toxin (DT) binds to the surface of cells expressing the heparin-binding epidermal growth factor-like growth factor (HB-EGF) precursor. DT-HB-EGF complex is internalized after endocytosis via clathrin-vesicles. Subsequently DT undergoes several posttranslational modifications resulting in a catalytically active toxin, called DT fragment A. This catalytically ribosylates elongation factor-2 (EF-2) leading to inhibition of protein synthesis with subsequent cell lysis and/or induction of apoptosis [37-40]. Like DT, Pseudomonas exotoxin A is also known to catalytically ribosylate EF-2 and thus leading to inhibition of protein synthesis. Extremely high cytotoxicity of this toxin with a lethal dose of 0.3 μg after i.v. injection in mice makes it a potential candidate for targeted cancer therapy [41]. *Clostridium perfringens *type A strain, the causative agent of gastroenteritis, produces *Clostridium perfringens *enterotoxin (CPE). The C-terminal domain of CPE is responsible for high affinity binding to the CPE receptor (CPE-R) and the N-terminal is assumed to be essential for cytotoxicity [42,43]. Studies have shown that purified CPE exerts an acute cytotoxic effect on pancreatic cancer cells and led to tumor necrosis and inhibition of tumor growth *in vivo*. It is being investigated for colon, breast and gastric cancers. Moreover, before evaluating CPE for systemic cancer therapy, its long term efficiency and lack of toxicity *in vivo *need to be demonstrated [44-46]. A recent study has demonstrated for the first time that botulinum neurotoxin (BoNT) briefly opens tumour vessels, allowing more effective destruction of cancer cells by radiotherapy and chemotherapy. It has been proposed that BoNTs act by an effect on the tumor microenvironment rather than by a direct cytotoxic effect on tumor cells [47]. Some bacterial toxins (alfa-toxin from *Stapylococcus aureus*, AC-toxin from *Bordetella pertussis*, shiga like toxins, and cholera toxin) are presently being studied on two cell lines, mesothelioma cells (P31) and small lung cancer cells (U-1690). Preliminary results with AC-toxin showed increasing cytotoxicity with increasing dose of AC-toxin in both cell lines and the toxin markedly increased apoptosis. However, cholera toxin did not induce apoptosis [34].

### Bacterial toxins conjugated to ligands

Protein toxins such as Pseudomonas exotoxin, diphtheria toxin, and ricin may be useful in cancer therapy because they are among the most potent cell-killing agents. Although they are very lethal yet for therapeutic efficacy these toxins need to be targeted to specific sites on the surface of cancer cells. This process is accomplished by eliminating binding to toxin receptors by conjugating the toxins to cell-binding proteins such as monoclonal antibodies or growth factors. These conjugates bind and kill cancer cells selectively thus sparing normal cells, which don't bind the conjugates. A wide variety of DT ligands such as IL-3, IL-4, granulocyte colony stimulating factor (G-CSF), transferrin (Tf), EGF and vascular endothelial growth factor (VEGF) have been studied for targeted tumors [38]. The transferrin-DT conjugate (Tf-CRM 107) and DT-EGF have reached the stage of clinical trials in patients of brain tumor and metastatic carcinomas respectively [48]. Similarly a large variety of antibodies and ligands to surface antigens overexpressed in different tumors have been conjugated to PE. Important ones tested in clinical trials are IL-4, IL-13, monoclonal antibody-recognizing a carbohydrate antigen Lewis Y, reacting with metastatic adenocarcinoma cells (Mab B3) and transforming growth factor (TGF-α) [49].

Another approach is to produce genetically modified or recombinant toxins. This is achieved by deleting the DNA coding for the toxin binding region and replacing it with various complementary DNA encoding other cell-binding proteins has been possible to make chimeric toxins that kill cells on the basis of the newly acquired binding activity. The ability to make these chimeras may be useful in designing future toxin-based anticancer therapies. For targeted DT therapy, deletions within the DT-receptor binding domain (amino acid residues 390-535) or targeted mutations of the critical HB-EGF precursor binding loop (amino acid residues 510-530) have been used [38,50]. Recently, a recombinant interleukin-4-Pseudomonas exotoxin (IL4-PE) for therapy of glioblastoma has been developed. *In vivo *experiments with nude mice have demonstrated that IL4-PE has significant antitumor activity against human glioblastoma tumor model. Intratumor administration of IL4-PE is being investigated for the treatment of malignant astrocytoma in a phase I clinical trial [51].

## Bacteria as immunotherapeutic agents

Immunotherapy for cancer offers great promise as an emerging and effective approach. Since tumors are immunogenic, the immunotherapeutic strategy employs stimulation of the immune system to destroy cancerous cells. But the major hurdle is the ability of tumors to escape the immune system due to development of tolerance as they are weakly immunogenic and sometimes body takes them as self antigens. Thus one of the novel immunotherapeutic strategies employs bacteria to enhance the antigenicity of tumor cells [52]. Attenuated but still invasive, *S. typhimurium *has been reported to infect malignant cells both *in vitro *and *in vivo*, thereby triggering the immune response. Attenuated *S. typhimurium *has demonstrated successful invasion of melanoma cells that can present antigenic determinants of bacterial origin and become targets for anti-Salmonella-specific T cells. However, better outcomes were achieved after vaccinating tumor bearing mice with *S. typhimurium *before intratumoral Salmonella injection [53]. Genetically engineered attenuated strains of *S. typhimurium *expressing murine cytokines have exhibited the capacity to modulate immunity to infection and have retarded the growth of experimental melanomas. Results have suggested that IL-2 encoding *Salmonella *organisms are superior in suppressing tumor growth as compared to the parental noncytokine-expressing strain [54]. Tumour antigen DNA sequences have been introduced into bacteria too such as Salmonella and Listeria, resulting in protective immunity in animal models. A xenogenic DNA vaccine encoding human tumor endothelial marker 8 (TEM8) carried by attenuated S. typhimurium has been reported to generate TEM8-specific CD8 cytotoxic T-cell response after oral administration. Suppression of angiogenesis in the tumors alongwith protection of mice from lethal challenges against tumor cells and reduced tumor growth support the potential of antiangiogenesis immunotherapy [55]. *C. novyi *has been reported to induce massive leukocytosis and inflammation. Furthermore, the antitumour effects of inflammation are well known too. Systemic administration of *C. novyi-NT *spores destroys adjacent cancer cells and triggers an inflammatory reaction by producing cytokines such as IL-6, MIP-2, G-CSF, TIMP-1, and KC that attract inflammatory cells i.e. neutrophils followed by monocyte and lymphocytes. The inflammatory reaction restricts the bacterial infection and directly contributes to the destruction of tumour cells through the production of reactive oxygen species, proteases, and other degradative enzymes. And finally it stimulates a potent cellular immune response leading to destruction of residual tumour cells. A phase I clinical trial combining spores of a *C. novyi-NT *with an antimicrotubuli agent has been initiated [52]. Because of its ability to stimulate strong innate and cell-mediated immunity, recombinant forms of the facultative intracellular bacterium, *Listeria monocytogenes*, have been used as vector for cancer vaccine. A recombinant *Listeria monocytogenes *vaccine strain (Lm-NP) expressing nucleoprotein (NP) from influenza strain A/PR8/34 has shown great therapeutic potential pre-clinically by regressing growth of macroscopic tumors of all types. Treatment with another recombinant listerial strain Lm-LLO-E7 has demonstrated effective cure of the majority of tumor bearing mice. And clinical trials are currently underway for the use of Lm-LLO-E7 as a cancer immunotherapeutic for cervical cancer [56]. An attenuated *Listeria monocytogenes *(LM)-based vaccine expressing truncated listeriolysin O (LLO) has demonstrated the eradication of all metastases and almost the entire primary tumor in the syngeneic, aggressive mouse breast tumor model 4T1 [57]. High efficacy of a Listeria-based Recently, a recombinant strain of attenuated *S. typhimurium *expressing a gene encoding LIGHT, a cytokine known to promote tumor rejection has been reported to inhibit growth of primary tumors, as well as the dissemination of pulmonary metastases, in various mouse tumor models employing murine carcinoma cell lines in immunocompetent mice. Antitumor activity was achieved without significant toxicity [58]. The cell wall skeleton of Mycobacterium bovis Bacillus Calmette-Guérin (BCG-CWS) has been used as an effective adjuvant for immunotherapy of a variety of cancer patients [59]. Recently it has been demonstrated that BCG/CWS has a radiosensitizing effect on colon cancer cells through the induction of autophagic cell death. *In vitro *as well as *in vivo *studies have revealed that BCG/CWS in combination with ionizing radiation (IR) is a promising therapeutic strategy for enhancing radiation therapy in colon cancer cells [60]. All these findings indicate the promising potential of nonvirulent bacteria as cancer immunotherapeutic agents.

## Bacterial spores

The majority of all the anaerobic bacteria discussed so far can form highly resistant spores which allow them to survive even in oxygen-rich conditions, although they cannot grow or multiply there. But once they meet favourable conditions, such as the dead areas inside tumors, the spores can germinate and the bacteria thrive, making them ideal to target cancers. Spores of genetically modified strain, *C. novyi*-*NT*, devoid of the lethal toxin have shown targeted action without any systemic side effects. Marked lysis of tumor tissues in mice receiving an intratumoral injection of *C. histolyticum *spores was observed. The same phenomenon was observed in mice injected intravenously with spores of *C. sporogenes*. In addition, Clostridium was detected only in tumors and not in normal tissues of mice receiving an intravenous injection of bacteria [61]. Pharmacologic and toxicologic evaluation of *C. novyi-NT *spores found that spores were rapidly cleared from the circulation by the reticuloendothelial system. No clinical toxicity was observed in healthy mice or rabbits even after large doses. However, in tumor-bearing mice, toxicity appeared related to tumor size and spore dose which is well as in case of any bacterial infection [62]. Bacterial spores have also been exploited as delivery agents for anticancer agents, cytotoxic peptides, therapeutic proteins, and as vectors for gene therapy. A summary of relevant clinical trials using bacteria is shown in Table 1.

**Table 1:** Important clinical trials involving bacterial intervention in cancer

Intervention (Number compound)	Clinical trial phase	Present status	Disease conditions
VNP20009	Phase I	Completed	Advanced or metastatic solid tumors [64]

TAPET-CD	Phase I	Completed	Head, neck and esophagus cancer [64]

Tf-CRM 107	Phase I	Ongoing	Brain entral nervous system tumors [48]

IL4-PE	Phase I	Ongoing	Brain entral nervous system tumors [51]

IL13-PE	Phase I	Ongoing	Malignant Glioma, Glioblastoma Multiforme, Anaplastic Astrocytoma, Anaplastic Oligodendroglioma and Mixed Oligoastrocytoma [49]

VNP20009- A genetically modified strain of *S. typhimurium*, TAPET-CD- Tumour amplified protein expression therapy using VNP20009 which expresses an *E. coli *Cytosine deaminase, Tf-CRM 107- Transferrin-Diptheria toxin conjugate, IL4-PE- Interleukin-4-Pseudomonas exotoxin, IL13-PE- Interleukin-13-Pseudomonas exotoxin.

## Problems with bacterial therapy

A major problem with using bacteria as anti-cancer agents is their toxicity at the dose required for therapeutic efficacy and reducing the dose results in diminished efficacy. Moreover, systemic infection of bacteria is rather inconvenient and carries higher risk of obvious toxicity. Furthermore, even removal of the toxin genes like in COBALT therapy led to ~15-45% mortality in mice [13]. Another major problem is incomplete tumor lysis i.e. bacteria don't consume all parts of the malignant tissue thus necessitating the combination of therapy with chemotherapeutic treatments. A more difficult problem is that of treating small non-necrotic metastases of large primary tumors as metastasis is the major cause of mortality from cancer. Because of small hypoxic regions of these metastases, targeting by bacteria is difficult. In case of bacteria based vector therapy the major hurdle is the inaccessibility because most of the times an intratumoural injection is required [63]. Another major concern regarding bacterial therapy is the potential for DNA mutations i.e. any loss of functionality due to mutations may lead to wide variety of problems like failure of therapy or exaggerated infection. Although some of the safety concerns have been solved with the recombinant DNA technology yet demands further development.

## Conclusion

Different applications of bacteria have been investigated so far as cancer treatment modalities. Live, attenuated bacteria as antitumor agents and vectors for gene-directed enzyme prodrug therapy have emerged as potential strategies. VNP20009 and TAPET-CD have been investigated successfully in Phase 1 clinical trials in cancer patients. Chimeric toxins are also being investigated as future toxin-based anticancer therapies. IL-4 fused with Pseudomonas exotoxin is in Phase I clinical trials in patients with glioblastoma. Further investigation and developments in these studies will add a new dimension to cancer treatment.

## Future directions

Currently bacteria have shown promising and significant potency in eradicating established tumors found in pre-clinical mouse tumor models. However the successful translation of these pre clinical strategies into clinical practice will depend on the outcome of clinical trials. Amongst all these, anaerobic bacteria vector-mediated cancer therapy and immunotherapy are very promising. But since cancer is a multifactorial disease no single therapy is completely suitable for it. A combination of recombinant DNA technology along with immunotherapy applied to the anaerobic bacteria will serve as the foundation for the multimodality therapeutic strategies for cancer.

## Abbreviations

AMP: Adenosine monophosphate; BCG: Bacillus Calmette-Guerin; BoNT: Botulinum neurotoxin; CMV: Cytomegalovirus; CD: Cytosine deaminase; CPG2: Carboxypeptidase G2; CDTs: Cytolethal distending toxins; CNF: Cytotoxic necrotizing factor; Cif: Cycle inhibiting factor; CPE: *Clostridium perfringens *enterotoxin; CPE-R: CPE receptor; COBALT: Combination bacteriolytic therapy; DCA: Dichloroacetate; DT: Diphtheria toxin; DNA: Deoxyribonucleic acid; EPEC: Enteropathogenic; EHEC: Enterohaemorrhagic; EF2: Elongation factor-2; EGF: Epidermal growth factor; 5FC-5-fluorocytosine; 5FU: 5-fluorouracil; G-CSF: Granulocyte colony stimulating factor; HAMLET: Human alpha-lactalbumin made lethal to tumor cells; HB-EGF: Heparin-binding epidermal growth factor-like growth factor; HSV: TK: Herpes simplex virus thymidine kinase; hGM-CSF: Human granulocyte-macrophage-colony stimulating factor; hIL-12: Human interleukin-12; IL-3: Interleukin-3; IL-4: Interleukin-4; IL4-PE: Interleukin-4-Pseudomonas exotoxin; Mab: Monoclonal antibody; mIL-12: Murine interleukin 12; mGM-CSF: Murine granulocyte-macrophage-colony stimulating factor; NR: Nitroreductase; TNF-α: Tumour necrosis factor α; TAPET: Tumour Amplified Protein Expression Therapy; Tf: Transferrin; Tf-CRM 107: Transferrin-DT conjugate; TGF-α: Transforming growth factor; VEGF: Vascular endothelial growth factor.

## Competing interests

The authors declare that they have no competing interests.

## Authors' contributions

SP carried out the literature survey and drafted the manuscript. RJ participated in literature survey. DS and AP provided the additional inputs, helped in sequence alignment and participated in proof-reading. BM conceived the idea and provided inputs for the design and final edition of the article. BK provided the relevant upcoming information related to the article and participated in the edition of the manuscript. All authors have read and approved the final manuscript.
